# Biopolymers-Based Macrogels with Applications in the Food Industry: Capsules with Berry Juice for Functional Food Products

**DOI:** 10.3390/gels10010071

**Published:** 2024-01-18

**Authors:** Roxana Elena Gheorghita, Ancuta Veronica Lupaescu, Anca Mihaela Gâtlan, Dadiana Dabija, Andrei Lobiuc, Oana Camelia Iatcu, Amelia Buculei, Alexandru Andriesi, Adriana Dabija

**Affiliations:** 1 College of Medicine and Biological Sciences, Stefan cel Mare University of Suceava, 13 University Street, 720229 Suceava, Romania; roxana.puscaselu@usm.ro (R.E.G.); ancuta.lupaescu@usm.ro (A.V.L.); andrei.lobiuc@usm.ro (A.L.); oana.iatcu@usm.ro (O.C.I.); 2 Suceava-Botoșani Regional Innovative Bioeconomy Cluster Association, Airport Street 1, 720134 Suceava, Romania; 3 Faculty of Food Engineering, Stefan cel Mare University of Suceava, University Street 13, 720229 Suceava, Romania; ameliab@fia.usv.ro (A.B.); adriana.dabija@fia.usv.ro (A.D.); 4 SC Natur Logistics SRL, 720043 Suceava, Romania; 5 Faculty of Economics, Administration and Business, Stefan cel Mare University of Suceava, Univeristy Street 13, 720229 Suceava, Romania; dabija.dadiana@gmail.com; 6 SC Andy Star SRL, 727011 Suceava, Romania; contact@aroniabio.ro

**Keywords:** chokeberry, sea buckthorn, blueberry, yogurt, sodium alginate

## Abstract

The present study focused on the development of gel-based capsules from sodium alginate and the fresh juice from different berries: chokeberry, sea buckthorn, and blueberry. Obtained through the extrusion method, the macrocapsules were added into yogurt, a well-known and consumed dairy product. In order to establish the changes that can occur for the food product, the samples were tested over 7 and 15 days of storage in refrigeration conditions. According to the results, the antioxidant activity increased during storage and gels can represent a good option for bioactive substances’ encapsulation. Sensorial analysis performed indicated that consumers are open to consuming yogurt berry capsules and, according to the results observed in the scientific literature, they no longer rejected the product due to the bitterness and sourness of sea buckthorn or aronia. Sea buckthorn capsules were brighter (L*) than chokeberry and blueberry capsules due to carotene content and dark colors. Minimal diameter variations and small standard deviations (SD = 0.25/0.33) suggest that extrusion methods and the Caviar box are good for gel capsule development. Yogurt luminosity varied with capsules; control had the highest, followed by sea buckthorn yogurt. Samples with chokeberry and blueberry (dark) capsules had lower luminosity. Over 8 and 15 days, luminosity slightly decreased, while a* and b* (hue and saturation) increased. Post-storage, the sample with chokeberry capsules showed a light purple color, indicating color transfer from capsules, with increased antioxidant activity. Differences between the samples and control were less pronounced in the sample with sea buckthorn capsules. Values for color differences between yogurt samples during the storage period revealed the most significant difference during the first storage period (day 1–8), with blueberries showing the lowest difference, indicating the stability of the blueberry capsules’ wall during storage.

## 1. Introduction

Dairy products, enjoyed globally, offer diverse options for all ages. Yogurts, acknowledged as functional dairy, contain prebiotics and probiotics. The food industry continually enhances and introduces fortified products with improved features. Efforts to increase durability and efficiency include encapsulation due to the natural fermentation process and sensitivity of bioactive substances. Encapsulation involves protecting a bioactive or sensitive substance by enclosing it in a coating. The materials used must be food-grade for safe consumption in food products or biodegradable to meet sustainability goals. The coating’s crucial role is to effectively shield the contents from external factors [1]. Due to their excellent properties and regenerative nature, edible biopolymers have been used as materials for encapsulating bioactive substances.

Among the most widely used encapsulation materials are polysaccharides, and among them, sodium alginate has the ability to efficiently and easily encapsulate various types of substances: colorants, flavorings, probiotic strains, phenolic compounds, antimicrobial or antioxidant substances, enzymes, essential oils, vitamins, minerals, or other functional ingredients [2,3,4,5,6,7]. To fortify dairy products, various substances have been encapsulated in biopolymeric matrices. In their study, Silva et al. highlighted that encapsulating guarana seeds and *Lacticaseibacillus paracasei BGP-1*, when added to yogurt drink, promoted the stability of probiotics (7 log cfu g^−1^/7 °C/28 days storage) and phenolic compounds to an extent of approximately 88%. This process prevented post-fermentation and eliminated the bitter taste of guarana seed extract [8]. Other studies highlighted the positive effect of natural substances encapsulated and added into the dairy products, such as the increased stability of pH and acidity during the storage period, reduced syneresis, and improved texture [9,10]. Encapsulation enhances polyphenol stability and gives better biodigestibility when different types of yogurt have been fortified with anthocyanins [11], rutin [12], or tamarillo [13]. Encapsulating anthocyanins in biopolymeric materials prolongs their bioavailability, offering a solution to regulatory concerns about red synthetic colorants in food. The European Union and the United States have restricted these colorants due to health concerns. The European Food Safety Authority limits dark colorant use, and the Chilean Food Health Regulation restricts it in products for children under 3 years of age [14]. Nowadays, the use of gels to encapsulate natural products that can be used as natural colorants or preserves is the best solution, not only for consumer health, but for the food industry as well.

In order to ensure sustainability and contribute to a circular economy, even by-products from the food industry have been incorporated into dairy products. Thus, yogurt with encapsulated carrot waste extract has a higher antioxidant activity than the control sample and can ensure a part of β-carotene recommended daily intake after consumption [15]. Same increased carotenoid and polyphenols retention have been observed when pepper waste capsules have been added to yogurt. Furthermore, the sensorial acceptability was higher than that of control sample and the lactic acid bacteria preservation was higher during storage [16]. 

According to research studies in recent years, consumers have refined their choices and focused their attention on functional foods with significant health benefits. Fruits such as aronia berries, blueberries, or sea buckthorn are recognized for their high antioxidant content and their beneficial effects on the microbiota [17,18,19]. 

Black chokeberry or aronia (*Aronia melanocarpa*) is a plant from the *Rosaceae* family that possesses high biological activity, being one of the richest plants in antioxidants, polyphenols, anthocyanins, with beneficial effects in frequent and modern pathologies such as dyslipidemia, hypertension or other cardiovascular disorders, diabetes or glucose metabolism disorders, obesity, and pro-inflammatory conditions. In vivo studies presented the beneficial effects of chokeberry in disorders and diseases associated with oxidative stress. Due to their high quantity of procyanidins, anthocyanins, and phenolic acids in the composition, aronia products may be useful as functional foods for these type of pathologies [20]. Other key benefits of aronia include preventing toxic effects, improving ulcerative colitis by reducing IL-6 and TNF-α levels, and treating influenza and urinary tract infections [21,22,23]. Clinical trials evidenced the effect of daily supplementation with chokeberry in reducing blood pressure and cholesterol [24]. Also, black chokeberry inhibits cancer cell growth by 24.7%, due to its rich content in polyphenols, antioxidant activity, and phenolic acids. In contrast, red and purple chokeberry stimulate cell proliferation by 23.2% and 27.2%, respectively [25]. The higher content in antimicrobial substances promotes *Aronia melanocarpa* as a naturally derived additive for maintaining the safety of food products against Gram-positive bacteria pathogens, such as *Bacillus cereus, Pseudomonas aeruginosa*, or *Staphylococcus aureus*. A preliminary study carried out by Hyeon-Kim et al. evidenced the capacity of aronia to inhibit and to avoid bacterial cell proliferation in yogurt and other dairy products [26].

Their high content in polyphenols attributes a bitter taste. For this reason, it is barely consumed as juices, syrups, wines, or as addition substances in food products [27]. 

Nonetheless, when using *Aronia melanocarpa* as a health supplement, it could demonstrate potential adverse effects, including diuretic and laxative effects due to its high-potassium and sorbitol content. Prolonged use may lead to anemia, tooth discoloration, and hypersensitivity due to anthocyanins [20]. 

Sea buckthorn (*Hippophae rhamnoides* L.) is a plant containing high amounts of biologically active substances, such as vitamins, minerals, polyphenols, carotenoids, phytosterols, amino acids, and fatty acids [28,29,30,31]. Used as a medicinal plant since ancient times, today it is consumed primarily for its beneficial properties on the cardiovascular system, microbiota, liver, or skin. Many research studies presented its antioxidant, antiviral, antimicrobial, anticancer, anti-inflammatory, anti-obesity, anti-hyperlipidemic, dermatological, and neuroprotective health benefits [29,32,33,34]. Due to its high content in flavonoids, daily consumption of 28 g of sea buckthorn for 90 days offers cardiovascular protection by addressing dyslipidemia, atherosclerosis, platelet aggregation, and reducing LDL and serum glucose levels [35]. Also, it has been demonstrated that sea buckthorn consumption for 3 months reduced hyperglycemia, insulin in blood, water intake, and sorbitol levels in the lens of eyes at Zucker diabetic fatty rats [36].

Widely used in various products like juices, yogurt, jams, beverages, tea, and bread, sea buckthorn enhances antioxidant properties and acidity when added to fermented drinks. In cheese, sea buckthorn fruits improve sensorial properties by protecting microbial cultures and promoting the growth of beneficial probiotic strain [37].

Despite the nutritional and benefits offered by sea buckthorn, the consumption is not as popular as it should be because of its unpalatable taste. The high content in total acid and malic acid, and its low sugar content make the product sour and astringent [38].

Blueberries (*Vaccinium ashei*) are recognized for their health benefits, such as high antioxidant and anticarcinogenesis activity, reduction in cardiovascular diseases, treatment of urinary tract disorders, microbiota modulation, or memory enhancement [39,40,41,42,43]. In a pilot study developed on 8–10-year-old children, a blueberry-based drink produced significant improvements in the delayed recall of a previously learned list of words [44].

Blueberries, rich in anthocyanins, have diverse uses in food, medicine, pharmacology, and cosmetics. They offer antioxidant benefits, enhance vision, reduce blood pressure, and help prevent conditions like diabetes and obesity. However, their bioavailability is influenced by factors such as temperature, pH, light, and oxygen exposure [45]. After ingestion, they are rapidly absorbed by the stomach and small intestine and, due to enzymes and intestinal flora, some of them are directly transformed into esters, glycosides, and polymers and are not absorbed by the small intestine [46]. Encapsulation is an effective method to protect and enhance the bioavailability of anthocyanins. This process improves stability during storage and prevents their release in the stomach [47]. Compared with freeze-dried juice, blueberry encapsulation presented better results in terms of release properties, ease of production, and potential applications [48]. Encapsulation proved to be efficient in prolonging the shelf life of anthocyanin extracted from blueberry, which maintains their properties even 115 days after development [49]. Encapsulation was a better method to preserve the antitumor properties. Tested in vitro, blueberry capsules improved the cell growth repression kinetics for the A549 cell line compared to the fresh fruits [50].

Fermenting blueberries or adding them to fermented products enhances anthocyanin bioavailability [51,52]. Alkaline conditions (pH > 8) have a negative effect and promoted the degradation due to the presence of quinoid base in the solution [53].

Based on all these scientific results, the present study focused on the development of biopolymer-based macrogels with aronia-, sea buckthorn-, and blueberry-encapsulated fresh juice and their addition to a common dairy product. Thus, yogurt with capsules was developed and stored for 15 days in refrigerated conditions. The tests, namely, physico-chemical evaluation and sensorial analysis, were performed on day 1, 8, and 15.

## 2. Results and Discussion

After development, the capsules and yogurt samples were physio-chemically evaluated and the results are presented in Table 1 and Table 2.

The luminosity (L*) of sea buckthorn capsules is higher than those of chokeberry and blueberry capsules due to the high content in carotenes and dark color of aronia and blueberry, respectively. As can be seen, the lowest values of the a* parameter are observed at capsules with blackberry in composition (chokeberry and blueberry).

The diameter of the samples presented low variations and the standard deviations are small (SD = 0.25/0.33). According to these results, the use of the extrusion method and Caviar box are good options for gel-capsule development.

The results presented in Table 2 present the yogurt stability during storage and low variations in pH, syneresis, and water-holding capacity. According to the results, the control sample was the most unstable and the yogurt with sea buckthorn presented the lowest pH variation (0.03 units) and the sample P3, with blueberry capsules in composition—the lowest variation in water-holding capacity (5.29 units). The same results were observed by Dabija et al., when 2% buckwheat and beetroot powder was added to yogurt, syneresis was lower and water-holding capacity was higher than the control sample [54]. Syneresis is correlated with the acidity of the samples, explaining the increase in syneresis over the shelf life for sample 2. Sea buckthorn, being a more acidic fruit compared to aronia and blueberries, contributes to this trend. For the other samples, P1 and P3, there is a decrease in syneresis values over the 15-day storage period of the yogurt samples. However, water-holding capacity (WHC) values show an increasing trend for all three samples during the storage period. This property is related to the protein content in the samples and the total amount of water that can be absorbed by the proteins, not significantly influenced by the type of microcapsules incorporated.

For all samples, the acidity has increased due to the continuous fermentation of the product. The higher reduction in pH was observed for yogurt with aronia capsules in composition. A slight decrease was observed at P2 sample, with sea buckthorn. Same results were observed by Tifrea et al. [55] and Guneac et al. [56] when they developed yogurt with sea buckthorn. The acidity of all samples was in the normal range, as found in the results presented by Brodziak et al. in their paper [57].

The luminosity of the yogurt sample was different, according to the capsules added. Thus, no matter the storage period, the highest luminosity can be observed in the control sample, followed by P2- yogurt with sea buckthorn in composition (Table 3). P1 and P3, with dark capsules added, presented lower values of luminosity. During 8, at 15 days, the luminosity slightly decreased, and the a* and b* parameters increased. The same result was observed by Najgebauer-Lejko et al. when they tested yogurt with sea buckthorn puree in composition [58].

**Figure 1:**
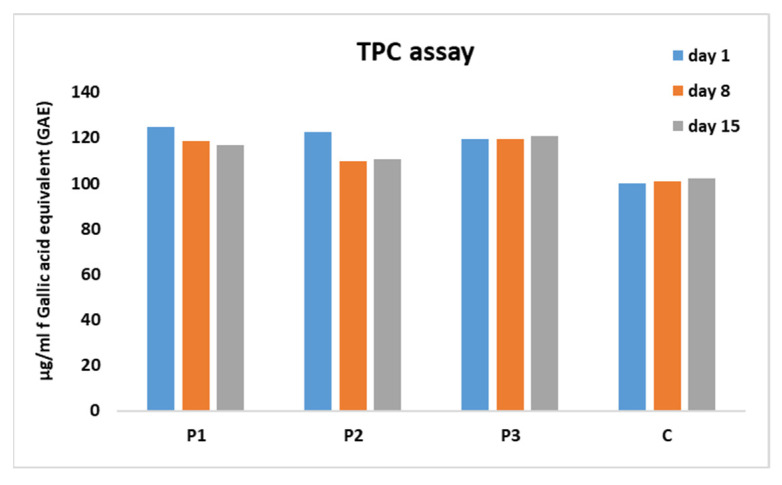
The temporal variation in total phenolic compound (TPC) content for microcapsule-enriched yogurt samples, expressed in μg/mL of Gallic acid equivalent (GAE). P1—yogurt enriched with chokeberry microcapsules; P2—yogurt enriched with buckthorn microcapsules; P3—yogurt enriched with blueberry microcapsules.

After storage, sample P1 exhibited a light purple color, even though the beads remained colored. The results indicate the transfer of color, implicitly anthocyanins, between the capsule and the product. This result is highlighted by the increase in antioxidant activity of the P1 sample during storage (Figure 1).

The differences observed between the samples and control are lower in the case of P2. According to Δ*Et* values, we can observe that the most notable difference was recorded for the first storage period (day 1–8), for samples P1, P2, and the control. P3, with blueberries in composition presented the lowest difference. The result evidenced the stability of the blueberry capsule wall during storage period.

### 2.1. Antioxidant Activity of Yogurt Samples

The antioxidant activity of microcapsule-enriched yogurts was assessed using the DPPH radical scavenging assay, a widely employed method for assessing the ability of compounds to counteract free radicals and provide hydrogen [59]. Chokeberry, sea buckthorn, and blueberry, which were used in the microcapsules, contain diverse chemical compounds known for their antioxidant properties [60]. Additionally, yogurt itself is recognized as a rich source of bioactive peptides with inherent antioxidant activity, particularly those generated during the fermentation process [61].

### 2.2. Total Phenolic Content and Antioxidant Activity of Yogurt Samples

Dietary polyphenols, recognized for their biologically significant roles as antioxidants, anticarcinogens, or antimutagens, have gained attention as potential nutraceuticals [62]. The evolution of total phenolic contents in samples over a 15-day storage period is depicted in Figure 1. The highest phenolic content was observed in the yogurt enriched with chokeberry microcapsules (P1). However, this content slightly decreased over time from 125 μg/mL GAE in day one, to 119 μg/mL GAE in day 8, and finally reaching 117 μg/mL GAE on the 15th day of storage. In contrast, the presence of blueberry microcapsules (P3) led to a slight increase in the TPC value over time, from 119 μg/mL GAE on day 1 to 121 μg/mL GAE on day 15. Similar results were observed in the control sample, with TPC values of 100 μg/mL GAE on day 1, 101 μg/mL GAE on day 8, and 101 μg/mL GAE on day 15. The presence of TPC may be attributed to phenols in milk, primarily derived from cattle feed [63]. However, potential interfering substances, such as aromatic amines, could react quantitatively with the Folin–Ciocalteu reagent [64]. Regarding the sample enriched with sea buckthorn (P2), the TPC level, initially 122 μg/mL GAE on the first day, experienced a significant decrease on the 8th day (110 μg/mL GAE) and remained constant until the 15th day. Similar results were observed in the case of yogurt enriched with black cumin [62] and yogurt prepared with combinations of heterofloral honey [65].

The antioxidant activity of yogurts enriched with microcapsules was evaluated using DPPH and ABTS radical scavenging assays. The DPPH assay, a commonly employed method for assessing the capacity of compounds to neutralize free radicals and donate hydrogen, relies on the reduction of the 2,2-diphenyl-1-picrylhydrazyl free radical by an antioxidant [60]. In contrast, the ABTS assay monitors the formation of the stable green–blue radical cationic chromophore, 2,2-azinobis-(3-ethylbenzothiazoline-6-sulfonate) (ABTS•+) [66].

Chokeberry, buckthorn, and blueberry, which were used in the microcapsules, contain diverse chemical compounds known for their antioxidant properties [60]. Additionally, yogurt itself is recognized as a rich source of bioactive peptides with inherent antioxidant activity, particularly those generated during the fermentation process [61].

**Figure 2:**
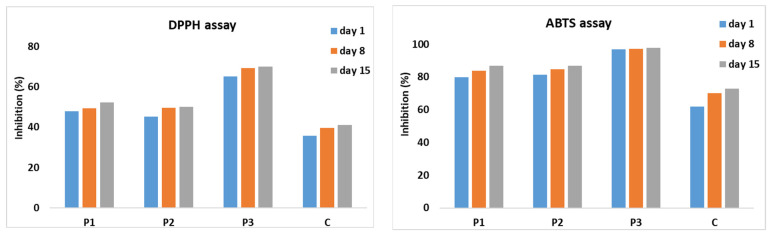
Evolution of antioxidant activity in time for control and microcapsule-enriched yogurt samples evaluated using DPPH (**left**) and ABTS (**right**). P1—yogurt enriched with chokeberry microcapsules; P2—yogurt enriched with buckthorn microcapsules; P3—yogurt enriched with blueberry microcapsules.

Figure 2 depicts the temporal changes in the antioxidant activity of microcapsule-enriched yogurt, with values measured during a 15-day storage period. As anticipated, the fermentation activity in the yogurt positively impacted the antioxidant activity of the control sample (C), with the percentage of inhibition showing a slight increase over time. However, a more pronounced enhancement was observed in the yogurt enriched with microcapsules, especially in the case of blueberry samples (P3). In this instance, the inhibition activity reached 97% in the ABTS assay and remained consistent throughout the observation period. This trend was also observed in the DPPH method, where the inhibition increased from 65% on day 1 to 70% on day 15. The rise in antioxidant capacity over time is also observable in samples enriched with aronia (P1) and sea buckthorn (P2), emphasizing the contribution of microcapsules to the overall antioxidant profile of yogurt.

### 2.3. The Rheological Properties of Yogurt Samples Characterization

**Figure 3:**
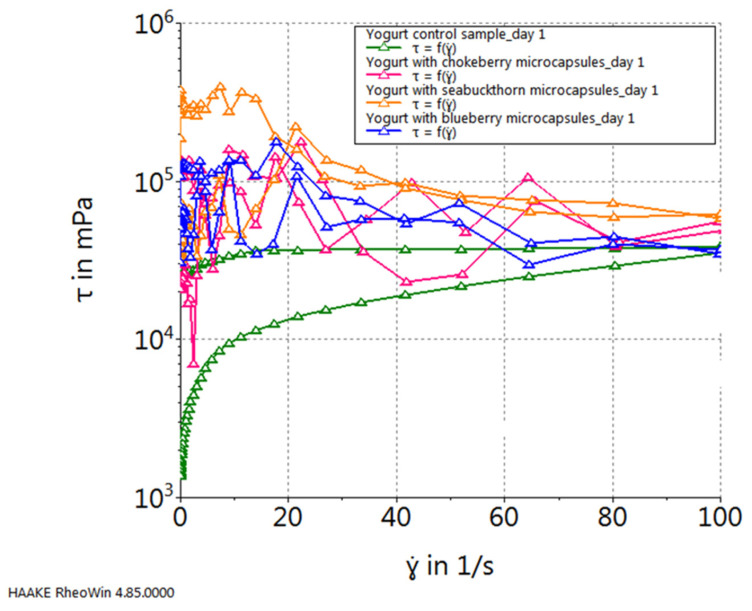
Flow curves of yogurt samples.

Regardless of the specific type of natural juice introduced into yogurt in the form of microcapsules, all yogurt samples exhibited non-Newtonian thixotropic behavior during flow. The thixotropic characteristics of the samples are evident from the graphical representation of flow curves (Figure 3), highlighting variations between the ascending and descending curves, indicative of gel breakage. These differences can be quantified by measuring the area between the flow curves: a larger area corresponds to a more pronounced thixotropic effect, reflecting greater gel breakage. The analysis indicates that there were no significant differences among the samples with various microcapsule additions. However, while examining yogurts with additional microcapsules in comparison to the control sample, a distinct trend emerges, characterized by the manifestation of discernible nonlinearities attributed to the rupture of microcapsules and the subsequent diffusion of the encapsulated juice within the yogurt volume.

**Figure 4:**
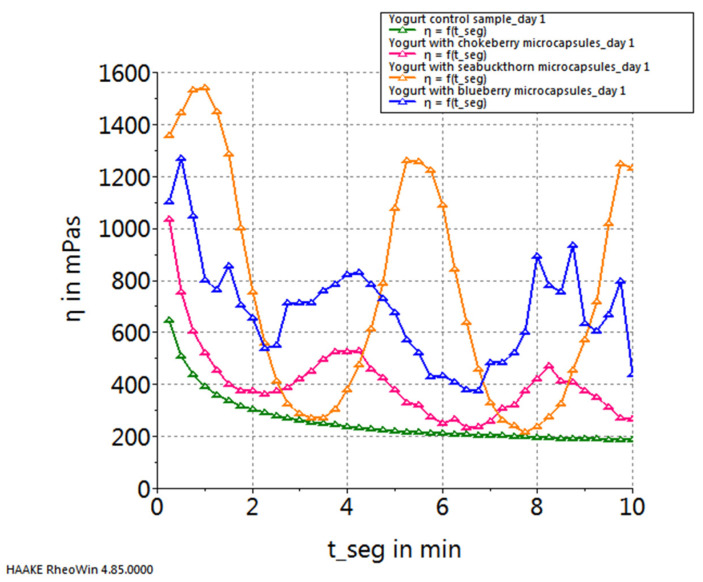
Viscosity curves of yogurt samples (day 1 of storage).

Figure 4 illustrates the temporal progression of viscosity in the samples on the first day of storage, at a constant shear rate of 100 s^−1^. Both initially and after undergoing a 10 min analysis, the samples exhibited consistent proportional relationships in terms of viscosity values. Consequently, the yogurt sample enriched with microcapsules containing sea buckthorn juice demonstrated the highest viscosity, succeeded by the yogurt sample incorporating microcapsules with blueberry juice, and subsequently, the yogurt sample with microcapsules infused with chokeberry juice, with the control sample of plain yogurt exhibiting the lowest viscosity. Furthermore, upon scrutinizing the viscosity curves depicted in Figure 3, a diminishing trend in viscosity is discernible relative to the duration of exposure to a shear rate of 100 s^−1^, marked by fluctuations in all samples correlating with the moment of microcapsule rupture. The yogurt sample featuring sea buckthorn microcapsules stands out, showcasing an initial uptrend in viscosity within the first minute, indicative of a harder microcapsule breakage. Subsequently, viscosity values fluctuate at levels significantly higher than those observed in yogurt samples with chokeberry or blueberry capsules—a phenomenon attributable to the elevated lipid content of sea buckthorn.

**Figure 5:**
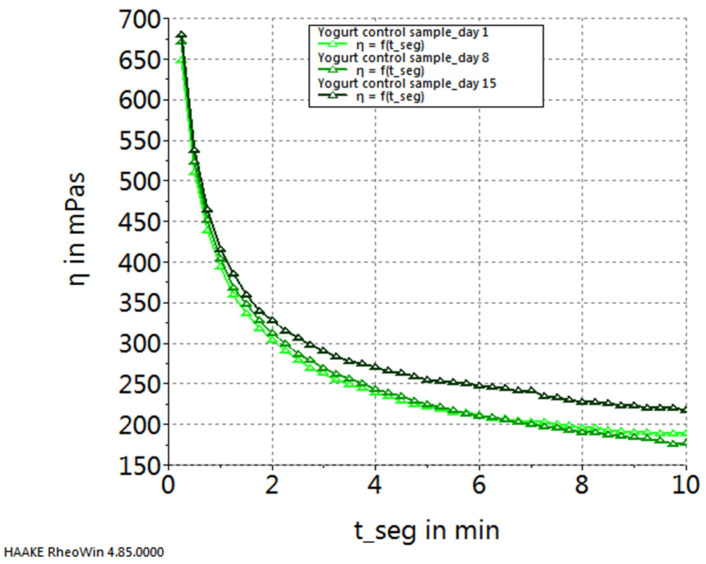
Variation in viscosity curves of control sample during storage.

**Figure 6:**
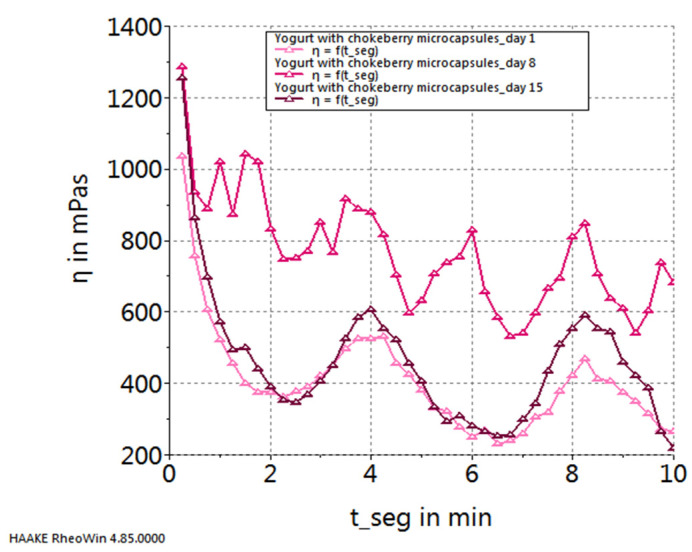
Variation in viscosity curves of yogurt with chokeberry capsules during storage.

**Figure 7:**
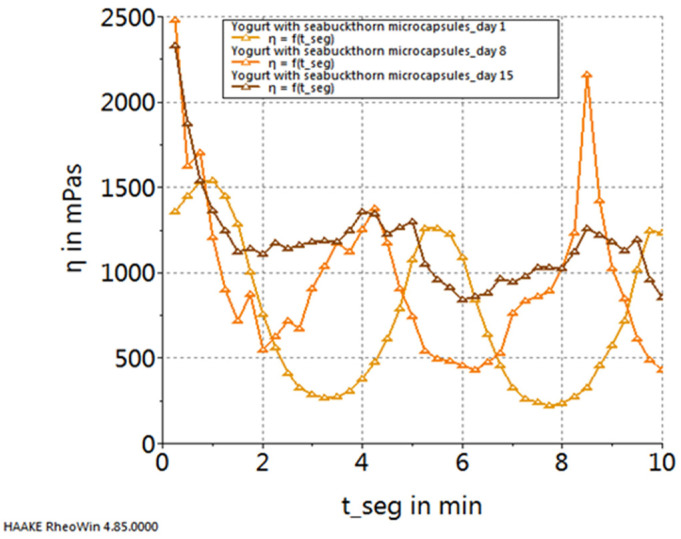
Variation in viscosity curves of yogurt with sea buckthorn capsules during storage.

**Figure 8:**
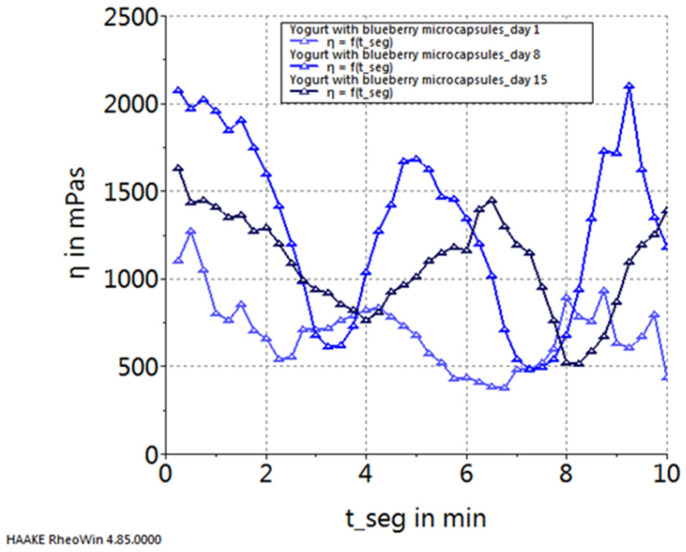
Variation in viscosity curves of yogurt with blueberry capsules during storage.

Figure 5, Figure 6, Figure 7 and Figure 8 depict the temporal variation in sample viscosity in relation to the duration of storage at refrigeration temperature (4 ± 0.3 °C). Three pivotal time points were considered: the initial day post-preparation, the 8th day of refrigeration, and the 15th day of refrigeration.

Upon analyzing the graphs illustrated in the figures, it is evident that all samples, both the control and those with the addition of microcapsules, exhibited the highest viscosity on the 8th day of storage. The exception to this trend is observed in the yogurt sample enriched with sea buckthorn microcapsules, where the highest viscosity was noted on the 15th day of storage.

**Figure 9:**
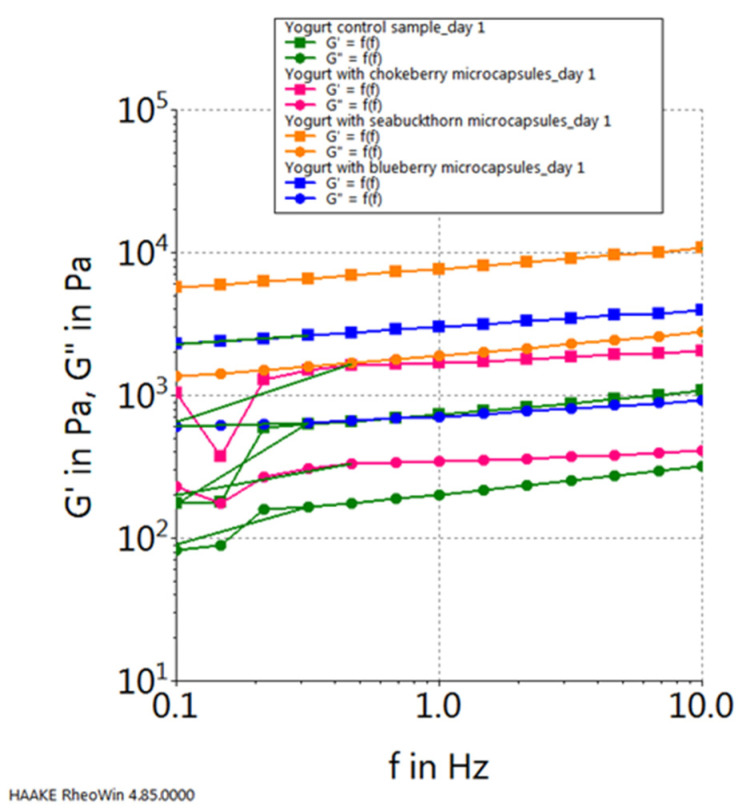
Viscoelastic properties of yogurt samples.

The mechanical spectra for the examined samples are shown in Figure 9. It can be observed that the consistency module values (G′) are slightly higher than the firmness module values (G″), and for both types of modules, they decrease in the following order: yogurt with sea buckthorn microcapsules > yogurt with blueberry microcapsules > yogurt with chokeberry microcapsules > plain yogurt.

**Figure 10:**
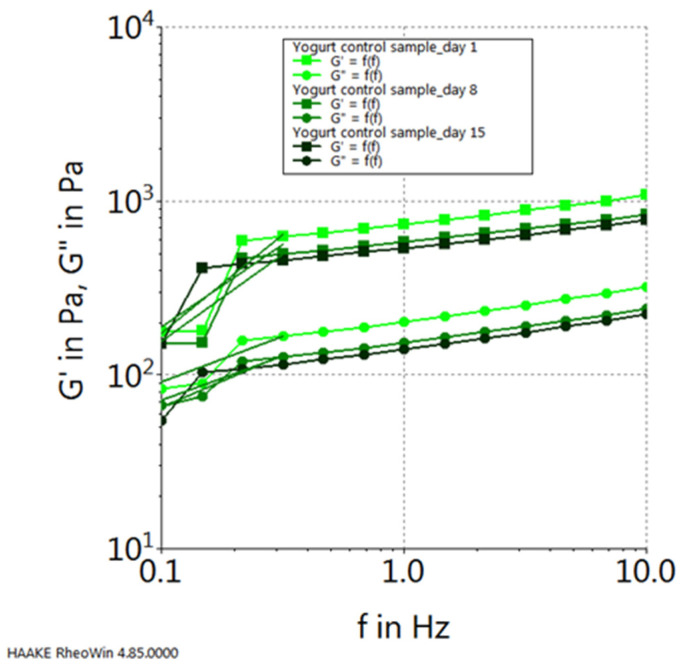
Viscoelastic properties of yogurt control sample during storage.

**Figure 11:**
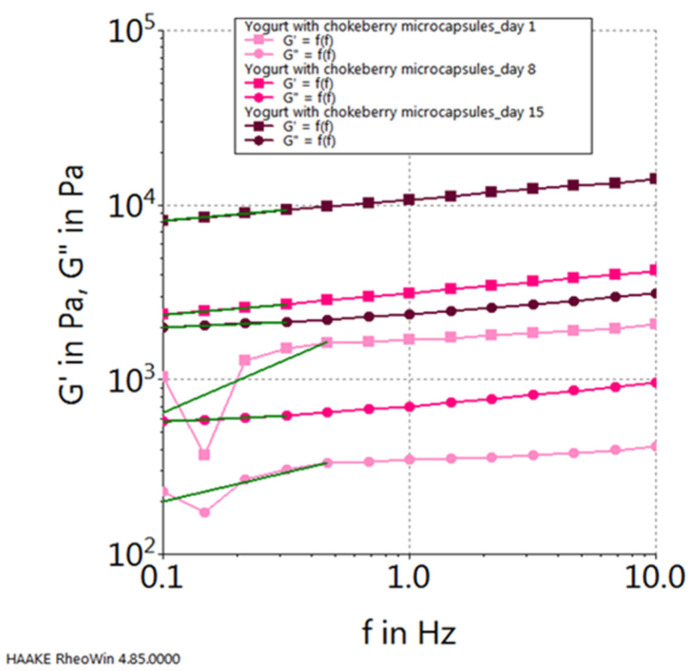
Viscoelastic properties of yogurt with chokeberry capsules during storage.

**Figure 12:**
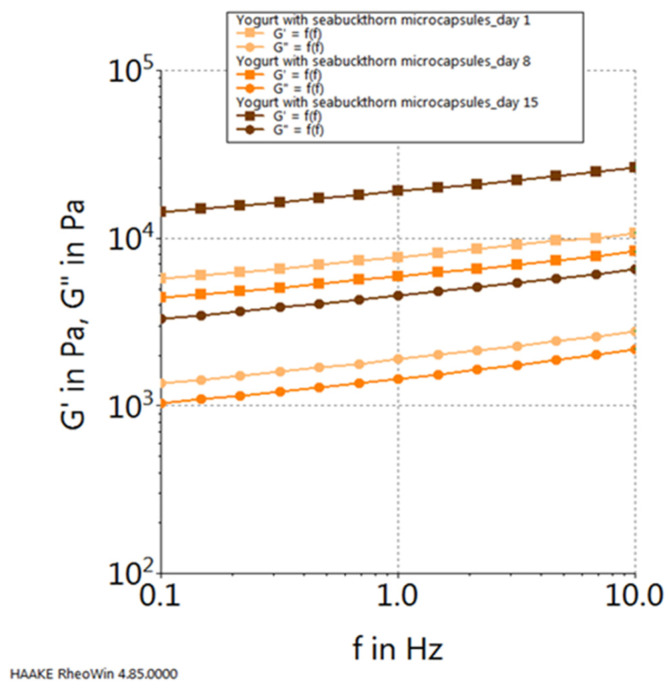
Viscoelastic properties of yogurt with sea buckthorn capsules during storage.

**Figure 13:**
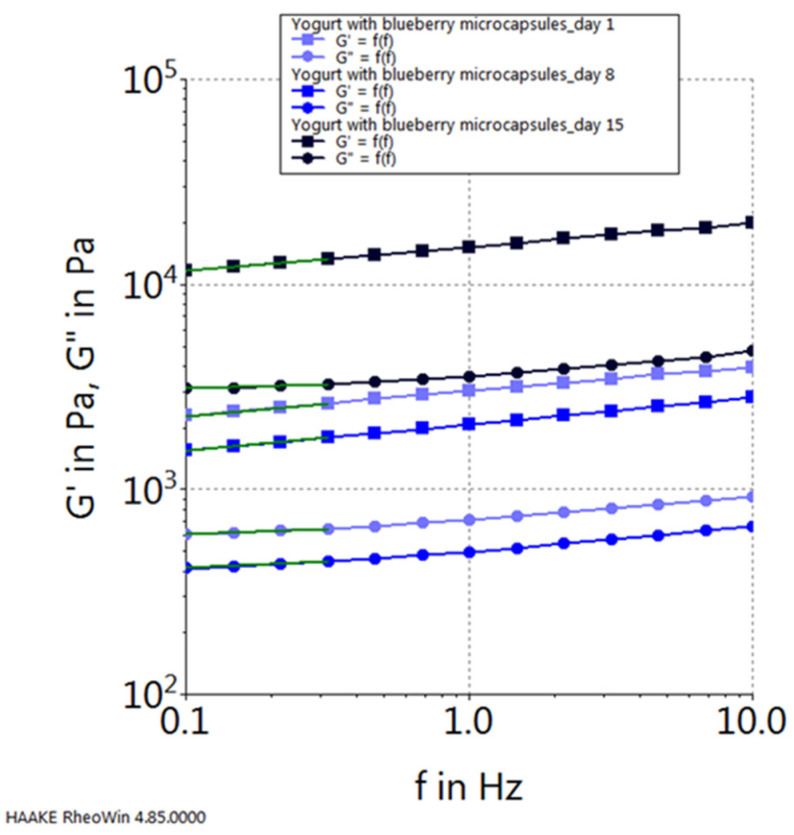
Viscoelastic properties of yogurt with blueberry capsules during storage.

Moreover, upon scrutinizing the progression of the elastic modulus (G′) and the viscous modulus (G″) for each sample, considering their dynamics throughout the storage period, the following observations emerge: in the case of the control sample (plain yogurt), both modules exhibit their peak values on the initial day of storage and reach their nadir on the final day of the 15-day storage period (refer to Figure 10). Conversely, for the yogurt sample containing chokeberry capsules, their progression follows an inverse pattern, with the highest values observed on the 15th day of storage and the lowest values recorded on the first day of storage (see Figure 11). In contrast, the yogurt samples featuring sea buckthorn microcapsules and those with blueberry microcapsules demonstrate their highest viscoelastic property values on the 15th day of storage, with the lowest values occurring in the middle of the storage period, specifically on the 8th day (refer to Figure 12 and Figure 13).

### 2.4. Sensory Evaluation

Criteria priorities. After inputting all questionnaire data from our 22 respondents and before proceeding to the aggregation of the judgments, the individual judgments made by each of the experts were checked for coherence. It was concluded that two of the experts were too inconsistent (consistency ratio > 0.20) and therefore, the final analysis was performed on the remaining 20-member group.

We further used the Spice Logic Analytic Hierarchy Process Software Group Decisions functionality for AIJ (aggregation of individual judgements) to compile the aggregated results from our panelists. Table 4 shows the pairwise comparisons, scoring weights, and relative priority of the criteria used for this study, with an overall consistency ratio from the aggregated results being satisfactory at 0.025. The calculated consistency ratio (CR) ensures that the judgments are reliable.

**Figure 14:**
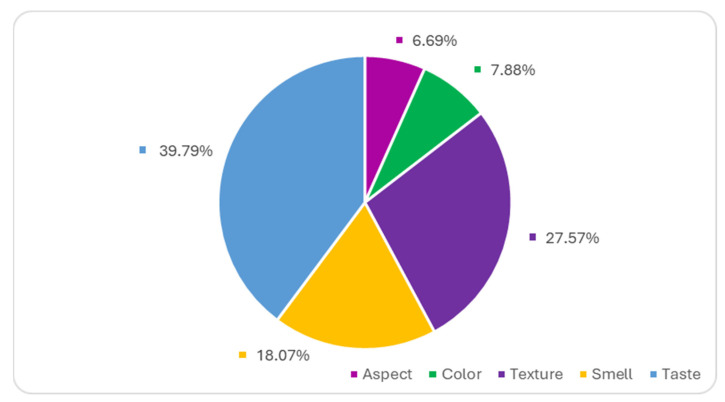
The importance of the criteria in ranking the yogurt samples obtained in the study.

Figure 14 highlights the weight of the importance of each sensory criterion in the total score awarded. The results obtained from the AHP method support the standard sensory analysis model, where taste (39.79%) and texture (27.57%) are the most important sensory properties, followed by smell (18.07%). While taste and texture are obviously the main characteristics that influence the consumer’s decision on which yogurt they prefer. The criteria for the aggregation of 20 individual judgements show that color and aspect are the least prioritized sensory characteristics when evaluating yogurt quality and will not influence the choice of the preferred yogurt.

Derive overall priorities of the alternatives. The Analytical Hierarchy Process (AHP) steps were implemented using Spice Logic version 4.2.6 Software for Windows Desktop to compute the criteria weights, criteria priority, and the overall priority for yogurt alternatives presented in this study. Based on the highest overall priority, the Analytical Hierarchy Process indicates that the best yogurt sample is P3—blueberry (63.50%), followed by P1—chokeberry (22.80%), and P2—sea buckthorn (12.60%).

**Figure 15:**
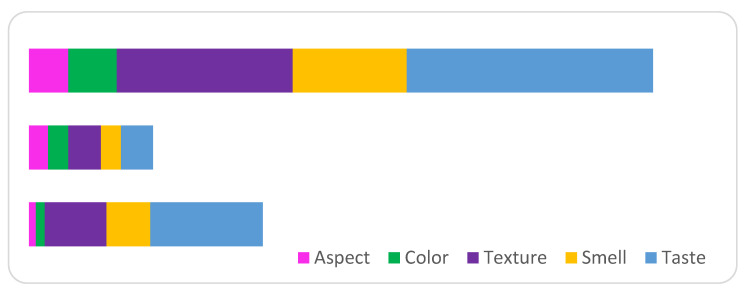
Classification of yogurt samples following sensory analysis using the AHP method.

Figure 15 and Table 5 summarize the ranking of the analyzed alternatives and the structure of the criteria behind the decision to rank the yogurt samples obtained by the tasters. Each column in the Figure shows the proportion of each criteria within the structure that were the basis behind the decision to rank the yogurt samples by the experts. The highest score was obtained by P3 (yogurt with blueberry capsules), followed by P1 (yogurt with chokeberry capsules), while P2 (yogurt with sea buckthorn capsules) obtained the lowest score for all sensory criteria analyzed. This ranking of yogurt samples confirms the results obtained through the standard sensory analysis method.

The sensitivity analysis showed the panelists marked all variables as insensitive. Therefore, the panelists did not show any variability that could drastically change the order of the yogurt samples’ prioritization, not even with the two closest criteria. In their study, Hyung Kim et al. examined the sensorial attributes (taste, color, flavor, texture, and overall acceptance) of yogurt, milk, and kefir with a chockeberry addition. According to their results, the milk with aronia received the lowest score, no matter the proportion of fruit added into the product. The highest scores were received by yogurt and kefir with 1% *Aronia melanocarpa*, with no differences when compared with the control sample, without chockeberry, which emphasizes the possibility of using this plant in fermentation dairy products. The yogurt with higher content of aronia (1.5%, 2%) into composition did not receive a good score. The main reason that could be taken into account is the astringent taste. According to the authors, a dairy product with more than 3% *Aronia melanocarpa* addition will be refused by almost all consumers [67].

The taste of yogurt with sea buckthorn presented the lowest value. Same results were observed by Laaksonen et al. when they tested the consumer acceptance of some berry fruits. According to 357 panelists, sea buckthorn was described as sour, bitter, and strong, but the acceptance could be positively influenced by their bright yellow color [68].

**Figure 16:**
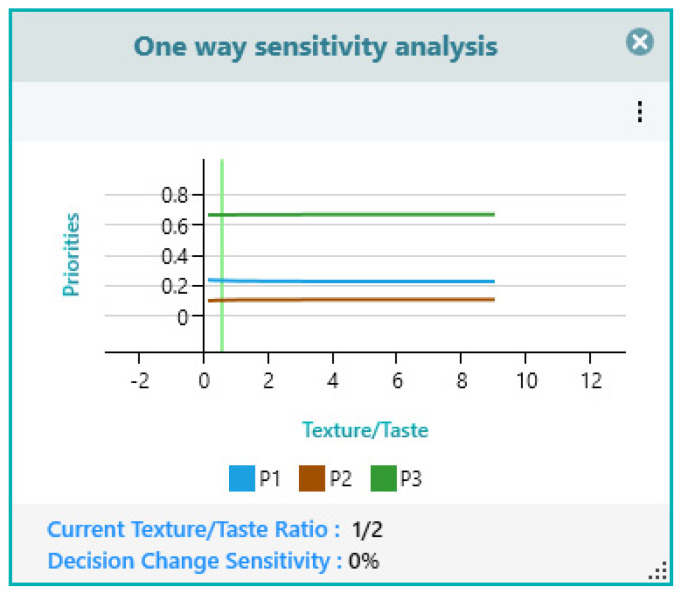
Criteria texture/taste sensitivity analysis.

In the present study, the taste and texture were analyzed side by side, and the results are presented in Figure 16.

## 3. Conclusions and Future Perspectives

The present paper evaluated the possibility of encapsulation berry fresh juices into sodium alginate and incorporation into a well-known dairy product. Despite their nutritional value, berry fruits and juices are consumed less because of their bitter taste. The encapsulation using sodium alginate through the extrusion method was a success in the macrocapsule development. According to our results, the yogurt with capsules maintained their antioxidant activity for a longer storage time. Sensorial evaluation classified the product in the following range: P3 (yogurt with blueberry capsules), followed by P1 (yogurt with chokeberry capsules), while P2 (yogurt with sea buckthorn capsules) obtained the lowest score for all sensory criteria analyzed. The sensitivity analysis showed the panelists marked all variables as insensitive and are open to consume the products. Future perspectives involve the testing in simulated gastrointestinal fluids to evaluate the bioavailability of yogurt with macrocapsules and their control release.

## 4. Materials and Methods

### 4.1. Materials

For the capsule development reagents, sodium alginate, DPPH, and calcium chloride solution were purchased from Sigma Aldrich Company, Romanian branch. Natural juice from organically certified aronia was provided by SC Andy Star SRL. According to the producer, 100 mL of chokeberry juice contains 0.11 g protein, 15.75 g carbohydrates (from which 6.49 g are natural sugars), 0.5 g fibers, and 0.0016 g salt. The energy value of the product is 64.88 kcal/100 mL juice.

Juice from organic sea buckthorn was provided by SC NATUR LOGISTICSSRL; 100 mL of juice contains 1 g fats (from which 0.2 g saturated fatty acids), 4 g carbohydrates (from which 4 g simple sugars), less than 0.8 g proteins and less than 0.01 g salt. The total energy value is 39 kcal/100 mL juice.

Blueberry juice was obtained in our laboratory by fresh fruit cold-pressing and its nutritional values are less than 0.5 g fat, 10 g carbohydrates, 9.2 g sugars, less than 0.5 g fibers, less than 0.5 g proteins, and less than 0.01 g salt. The energetic value of 100 mL of juice was 41 kcal.

For yogurt preparation we used fresh raw cow milk with 3.5% fat, 3% protein and 4.5% carbohydrates content, from our local producers. Lactic bacteria such as *Lactobacillus bulgaricus* and *Streptococcus thermophilus* were provided from SC Enzyme & Derivates SA Romania.

### 4.2. Development of Gel Macrocapsules and Yogurt

Using fresh juice as the liquid phase, 5% sodium alginate capsules were developed. Thus, aronia, sea buckthorn, and blueberries and sodium alginate were heated at 60 ± 3 °C for 20 min, under stirring conditions (550 rpm). Macrocapsules were obtained through extrusion method, using a Caviar box. The capsules were released into 2% calcium chloride solution, maintained for 3 min for coating development and rinsed in fresh water in order to eliminate the excess solution. Immediately after development, the capsules were used for yogurt preparation. The yogurt was prepared according to the technological scheme used in the industry: milk pasteurization (70 ± 5 °C/15 min), followed by cooling until 40 °C and adding the strains of microorganisms. In this step, we added 10% capsules as well. According to our previous study [69], the time it takes for the capsules’ addition into the yogurt preparation does not influence the final properties of food products. The mixtures were homogenized, dosed in 100 mL glass jars, and incubated at the same temperature until pH 4.6. After that, the yogurt was cooled and stored at 4 °C for further evaluation. Yogurt without capsules represents the control sample.

### 4.3. Color Evaluation

Color was evaluated through the CIELAB system, using a Konica Minolta CR 400 colorimeter, taking into account the parameters such as L*, a*, and b*. The L*-axis determines lightness, with a white object having an L* value of 100, and a black object having an L* value of 0. Achromatic colors, such as various shades of grey, are positioned along the L*-axis.

Chromatic or colors are characterized using two axes in the horizontal plane. The a*-axis represents the green–red spectrum, while the b*-axis extends from blue (−b*) to yellow (+b*). Each color finds representation as a color point (L*, a*, b*) within the color space, where L*, a*, and b* serve as the color coordinates for that point. The asterisk attached to *L*, *a*, and *b* signifies that this denotes the new color system, succeeding the older CIELAB system. Although the simplified notation of Lab values, without the * symbol, is often employed, the new system is universally accepted for color quantification [70].

Our results were noted as an average of five readings in different areas of food products. White plate parameters are L* = 94.39, a* = −0.31, and b* = 4.13.

The color variations between the control sample and yogurt with chokeberry capsules (*P1*), sea buckthorn capsules (*P2*), and blueberry capsules (*P3*) were evaluated using Formula (1):

(1) $$\Delta E=\sqrt{{\Delta L*}^{2}+{\Delta a*}^{2}+{\Delta b*}^{2}}$$

where Δ*Es* represents the total color differences between the sample color parameters and control samples. Δ*Et* represents the total color differences of the samples tested on the first day and after storage at 7 and 15 days.

### 4.4. Capsules Diameter

Capsule size was evaluated using an Yato electronic micrometer (Shanghai, China), with a precision of 0.002 mm. The result was expressed as an average of at least 10 readings of capsules.

The *Syneresis* (*S*) of yogurt samples was tested on day 1, 7, and 15. For syneresis, a 100 mL sample was placed into a funnel and filtered for 6 h. After that, the volume from funnel was noted and used to calculate, according to the following formulas:

(2) $$Syneresis\;\left(\%\right)=\frac{V1}{V2}\times 100$$

where *V*1 is the volume of the whey collected after drainage and *V*2 is the volume of the yogurt sample.

*Water-holding capacity (WHC)* was tested by centrifugation of 5 g yogurt at 4500× *g* for 15 min and 4 °C. The parameter was calculated according to Formula 3, where *W*1 is the whey mass after centrifugation and *W*2 is the mass of the initial sample (5 g).

(3) $$WHC\;\left(\%\right)=1-\frac{W1}{W2}\times 100$$

The pH was evaluated using a Metter Toledo pH-meter (Carl Roth, Karlsruhe, Germany) and the result was noted as the average of at least five readings in different areas of the product.

### 4.5. Determinations of Total Phenolic Compounds (TPC) and Antioxidant Activities (DPPH—2,2-Diphenyl-1-Picrylhydrazyl and ABTS)

The determination of total phenolic compounds (TPC) involved the use of Folin–Ciocalteu and sodium carbonate (Na_2_CO_3_) reagents. Total polyphenols were determined using the modified Folin–Ciocalteu procedure [71]. Initially, 2 g of yogurt samples underwent centrifugation at 12,000 rpm for 5 min at 4 °C. Subsequently, the supernatants were re-centrifuged under the same conditions. A mixture of 100 μL of yogurt extract and 100 μL of Folin–Ciocalteu’s solution (1 mol/L) was prepared, followed by the addition of 300 μL of 10% sodium carbonate solution after a 5 min incubation at room temperature. The resulting solution was kept at room temperature for 30 min. In the final step, 1 mL of distilled water was added to the mixture, and the absorbance was measured at 725 nm. Gallic acid served as a standard at concentrations of 60, 80, 100, 120, and 150 μg/mL. The results are expressed as μg/mL of gallic acid equivalent (GAE), calculated using the equation y = 0.0074x − 0.2742, with an R^2^ value of 0.99, where x represents the absorbance, and y denotes the gallic acid equivalent.

Determination of DPPH radical scavenging activity was used for the assessment of the antioxidant activity of yogurt samples [70]. Briefly, 100 μg of yogurt extract and 900 μL of 0.094 mM 2,2-diphenyl-1-picrylhydrazyl previously dissolved in methanol were incubated in the dark at room temperature for 1 h. The absorbance of the mixture was measured at 517 nm, and the results were given as inhibition percent using the following equation:

DPPH radical scavenging activity (%) = [(A_control_ − A_sample_)]/(A_control_)] × 100, (4)

where A_control_ is the absorbance of DPPH radical + methanol; A_sample_ is the absorbance of DPPH radical + sample extract.

Antioxidant activity by the ABTS method was performed using a colorimetric assay with the ABTS reagent (2,2′-Azinobis (3-ethylbenothiazoline-6-sulfonic acid) and potassium persulfate (K_2_S_2_O_8_) [72]. Briefly, 100 μL of yogurt extract was mixed with 900 μL of ABTS reagent. Absorbance was verified in a spectrophotometer at 734 nm after 6 min of incubation. Results were expressed as percentage inhibition ABTS radical scavenging activity using the following equation:

ABTS, (%) = [(A_control_ − A_sample_)]/(A_control_)] × 100, (5)

where A_control_ is the absorbance of ABTS radical + water; A_sample_ is the absorbance of ABTS radical + sample extract.

### 4.6. Assessment of the Rheological Properties of Yogurt Samples

The rheological attributes of yogurt samples were assessed using a state-of-the-art Modular Advanced Rheometer System (Thermo Haake Mars, Karlsruhe, Germany) equipped with a measuring system featuring a titanium geometry plate (40 mm in diameter, with a 1 mm gap). The measurements were conducted at a temperature of 8 °C, following a 10 min resting period for the samples. Varied shear stresses were applied to the samples to observe their tension and viscosity parameters. To construct flow curves, shear rates ranging from 0.02 to 100 s^−1^ and from 100 to 0.02 s^−1^ were applied. The analysis also encompassed evaluating the fluctuation in sample viscosity in relation to shear rate within the same interval. Distinct models were fitted to the viscosity curve of each sample to identify the most fitting model that captures the rheological characteristics. Furthermore, the viscosity of the samples was monitored over a 10 min period at a constant shear rate of 100 s^−1^, yielding 40 data points. Oscillatory rheological properties were examined through frequency dependency tests across a frequency range from 0.1 to 10 Hz. All parameter calculations were executed using RheoWin 4 Data Manager software (version 4.20, Haake). Each sample underwent three determinations for every rheological test [73,74].

### 4.7. Sensory Evaluation

Sensory evaluation is a widely used method in the food industry to evaluate yogurt samples and estimate overall acceptability. However, the application of sophisticated decision-making tools, such as the Analytical Hierarchy Process (AHP), in the sensory analysis of food and beverage industry remains relatively unexplored [75,76]. In the context of sensorial analysis of yogurt samples, the Analytical Hierarchy Process (AHP) model can be employed to systematically evaluate and prioritize the sensory attributes of different yogurt samples based on the preferences of experts or consumers.

Determine matrix criteria. For this AHP model, we first created a decision hierarchy that represents the main components of the problem. The sensory criteria were selected based on the industry standard for yogurt sensory analysis. In the context of this specific yogurt sensorial analysis, our matrix criteria included the following criteria:

1. Goal: Evaluate the overall sensorial quality of yogurt samples.
2. Criteria: Different sensory attributes such as aspect, color, texture, smell, and taste.
3. Alternatives: Three yogurt samples being analyzed: P1 (aronia), P2 (sea buckthorn), and P3 (chokeberries).

Pairwise comparisons of criteria. The next step in AHP methodology is to establish pairwise comparisons for each pair of elements in the hierarchy as shown in Table 6. This involves determining the relative importance or preference of one element over another. Experts or consumers can provide their judgments on the importance of each criterion concerning the others. A scale like Saaty’s scale (1–9) is commonly used for this purpose [77,78].

We asked a group of 22 expert panelists to taste our yogurt samples with a total of three samples per tasting: P1 (aronia), P2 (sea buckthorn), and P3 (blueberries). The panel consisted of 11 men and 11 women. Samples were presented to the tasters in random order and were coded through a blind test. Each of the group members was given a questionnaire to perform the pairwise comparisons required by the AHP method. Two sets of comparisons were made: those between tasting attributes and those between the yogurt samples.

**Figure 17:**
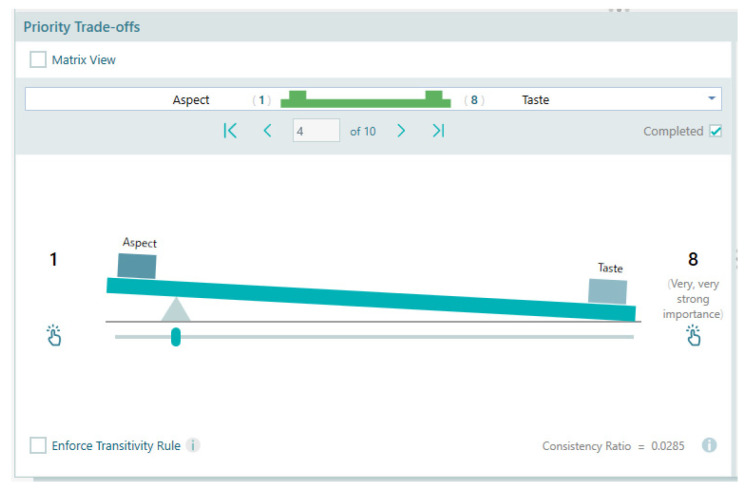
Criteria pairwise comparison example using Analytic Hierarchy Process Software 4.2.6 for Windows Desktop.

**Figure 18:**
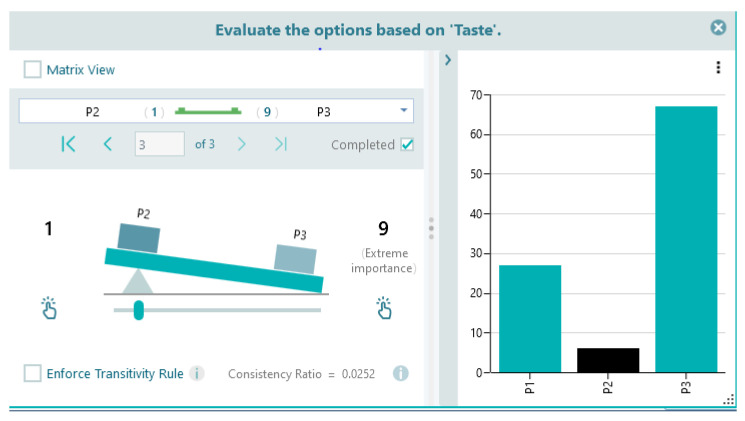
Alternatives pairwise comparison example using Analytic Hierarchy Process Software 4.2.6 for Windows Desktop.

Each criterion is evaluated separately. For example, to evaluate the quality of a yogurt, in the first line of the table, the panelist chooses whether the taste of the yogurt is much more important than its aspect (Figure 17). The data obtained through the questionnaires were later introduced in the Spice Logic Analytic Hierarchy Process Software version 4.2.6 for Windows Desktop [79]. This AHP software uses a straightforward and intuitive user experience to ascertain the pairwise comparison between criteria (Figure 17) and between alternatives (Figure 18), as seen below:

Statistical differences between samples were calculated with SPSS 20 software (SPSS Inc. Chicago, IL, USA).

## Figures and Tables

**Table 1:** Color and diameter evaluation of macrocapsules.

	Chokeberry Capsules	Sea Buckthorn Capsules	Blueberry Capsules	p1*	p2**	p3***
L*	28.89 ± 0.31	54.44 ± 0.32	28.65 ± 0.29	<0.001	0.726	<0.001
a*	1.53 ± 0.03	13.17 ± 0.08	1.21 ± 0.22	<0.001	0.303	<0.001
b*	0.82 ± 0.06	0.79 ± 0.14	0.78 ± 0.05	0.934	0.921	0.999
Diameter, mm	3.12 ± 0.33	3.27 ± 0.25	3.08 ± 0.03	<0.001	0.137	<0.001

p1*—statistical differences between chokeberry and sea buckthorn capsules; p2**—statistical differences between chokeberry and blueberry capsules; p3***—statistical differences between sea buckthorn and blueberry capsules.

**Table 2:** Yogurt evaluation for the two-week storage period.

	P1	P2	P3	C	p1*	p2**	p3***
pH							
Day 1	4.56 ± 0.02	4.52 ± 0.02	4.38 ± 0.02	4.34 ± 0.01	<0.001	<0.001	0.463
Day 8	4.52 ± 0.01	4.50 ± 0.01	4.40 ± 0.01	4.32 ± 0.01	0.001	0.002	0.036
Day 15	4.45 ± 0.01	4.49 ± 0.01	4.37 ± 0.01	4.31 ± 0.01	0.003	<0.001	0.021
pa	0.172	0.676	0.983	0.985			
pb	0.056	0.365	0.998	0.817			
pc	0.020	0.865	0.971	0.996			
Syneresis, %							
Day 1	47.00 ± 0.01	50.00 ± 0.01	53.50 ± 0.01	52.00 ± 0.01	0.004	0.070	0.069
Day 8	45.00 ± 0.01	50.80 ± 0.01	51.30 ± 0.01	46.00 ± 0.01	0.288	0.001	0.001
Day 15	44.00 ± 0.01	53.00 ± 0.01	50.50 ± 0.01	40.00 ± 0.01	0.008	<0.001	<0.001
pa	0.001	0.548	0.003	<0.001			
pb	0.002	0.037	<0.001	<0.001			
pc	0.005	0.010	0.037	<0.001			
Water-holding capacity, %							
Day 1	31.14 ± 0.01	38.17 ± 0.01	33.75 ± 0.01	35.84 ± 0.01	<0.001	<0.001	<0.001
Day 8	35.17 ± 0.01	41.12 ± 0.01	31.19 ± 0.01	34.12 ± 0.01	<0.001	<0.001	<0.001
Day 15	38.84 ± 0.01	44.76 ± 0.01	39.04 ± 0.01	30.86 ± 0.01	<0.001	<0.001	<0.001
pa	<0.001	<0.001	<0.001	<0.001	pa	<0.001	<0.001
pb	<0.001	<0.001	<0.001	<0.001	pb	<0.001	<0.001
pc	<0.001	<0.001	<0.001	<0.001	pc	<0.001	<0.001

P1—yogurt with chokeberry capsules; P2—yogurt with sea buckthorn capsules; P3—yogurt with blueberry capsules; C—control sample; p1*—statistical differences between P1 and C; p2**—statistical differences between P2 and C; p3***—statistical differences between P3 and C; pa—statistical differences between day 1 and day 8 of storage; pb—statistical differences between day 1 and day 15 of storage; pc—statistical differences between day 8 and day 15 of storage.

**Table 3:** Color variations between samples during storage period.

	P1	P2	P3	C
L*				
Day 1	71.92 ± 1.93	77.94 ± 0.64	74.70 ± 0.76	79.71 ± 0.08
Day 8	72.49 ± 0.29	78.47 ± 0.24	74.85 ± 0.46	78.74 ± 0.21
Day 15	73.22 ± 0.06	78.62 ± 0.29	74.04 ± 0.40	79.17 ± 0.47
a*				
Day 1	3.13 ± 0.24	1.47 ± 0.02	0.49 ± 0.09	−2.45 ± 0.03
Day 8	3.32 ± 0.13	1.50 ± 0.01	0.91 ± 0.04	−2.51 ± 0.02
Day 15	3.84 ± 0.02	1.54 ± 0.01	1.29 ± 0.19	−2.52 ± 0.02
b*				
Day 1	2.45 ± 0.19	8.22 ± 0.10	4.79 ± 0.39	8.68 ± 0.02
Day 8	3.81 ± 0.07	8.24 ± 0.02	4.73 ± 0.02	8.64 ± 0.02
Day 15	4.20 ± 0.02	8.29 ± 0.03	4.68 ± 0.41	8.56 ± 0.08
Δ*Es*				
Day 1	9.99	2.07	6.63	-
Day 8	7.94	1.11	5.74	-
Day 15	7.49	1.15	3.12	-
Δ*Et*				
Day 8–Day 1	1.48	0.53	0.45	0.99
Day 15–Day 8	0.83	0.16	0.89	0.43
Day 15–Day 1	2.58	0.71	1.04	0.55

Δ*Es*—color difference between yogurt with capsules and control sample; Δ*Et*—color differences between yogurt during storage period.

**Table 4:** Pairwise comparation between main sensorial parameters of samples.

	Aspect	Color	Texture	Smell	Taste	Priorities
Aspect	1	0.607	0.252	0.339	0.226	0.0669
Color	1.65	1	0.24	0.322	0.21	0.0788
Texture	3.97	4.16	1	2.04	0.473	0.2757
Smell	2.79	3.11	0.49	1	0.441	0.1807
Taste	4.43	4.77	2.11	2.27	1	0.3979
Consistency Ratio calculated as				0.025		

**Table 5:** Classification of yogurt samples following sensory analysis using the AHP method.

Option	Aspect	Color	Texture	Smell	Taste
P1	0.0072	0.0089	0.0631	0.0444	0.1146
P2	0.0195	0.0207	0.0332	0.0203	0.0325
P3	0.0402	0.0492	0.1794	0.1159	0.2508

**Table 6:** Example of questions received by each taster for evaluation of sensory properties.

From Your Point of View, Which Attribute Is More Important and to What Extent to Evaluate the QUALITY of a Yogurt?										
	EX	MF	F	MO	=	MO	F	MF	EX	
C1 Aspect	9	7	5	3	1	3	5	7	9	C2 Color
C1 Aspect	9	7	5	3	1	3	5	7	9	C3 Texture
C1 Aspect	9	7	5	3	1	3	5	7	9	C4 Smell
C1 Aspect	9	7	5	3	1	3	5	7	9	C5 Taste
C2 Color	9	7	5	3	1	3	5	7	9	C3 Texture
C2 Color	9	7	5	3	1	3	5	7	9	C4 Smell
C2 Color	9	7	5	3	1	3	5	7	9	C5 Taste
C3 Texture	9	7	5	3	1	3	5	7	9	C4 Smell
C3 Texture	9	7	5	3	1	3	5	7	9	C5 Taste
C4 Smell	9	7	5	3	1	3	5	7	9	C5 Taste

## Data Availability

The data presented in this study are openly available in article.
